# Supplementary material for: Cryo-EM structures of Aβ40 filaments from the leptomeninges of individuals with Alzheimer’s disease and cerebral amyloid angiopathy
Source: Acta Neuropathol Commun. 2023 Dec 4;11:191. doi: 10.1186/s40478-023-01694-8 (PMC10694933; doi:10.1186/s40478-023-01694-8)
Supplement: Supplementary file 1 — Additional file 1: Table S1. Cryo-EM data acquisition and structure determination. Table S2. Particle numbers of each data set. [file 40478_2023_1694_MOESM1_ESM.docx]

**SUPPLEMENTARY TABLE**

**Table S1. Cryo-EM data acquisition and structure determination.**

|  | **Aβ40 filaments (sarkosyl-extracted)** | |
| --- | --- | --- |
|  | **Type 2** | **Type 1** |
|  | (EMD-18508, PDB 8QN6) | (EMD-18509, PDB8QN7) |
| **Data acquisition** |  |  |
| Electron gun | CFEG | |
| Detector | Falcon4i | |
| Energy filter slit (eV) | 10 | |
| Magnification | 165,000 | |
| Voltage (kV) | 300 | |
| Electron dose (e^–^/Å^2^) | 40 | |
| Defocus range (μm) | -1.0 to -2.4 | |
| Pixel size (Å) | 0.744 | |
|  |  |  |
| **Map refinement** |  |  |
| Symmetry imposed | C2 | C1 |
| Initial particle images (no.) | 211,923 | |
| Final particle images (no.) | 81,140 | 32,218 |
| Map resolution (Å) | 2.4 | 2.7 |
| FSC threshold | 0.143 | 0.143 |
| Helical twist (°) | -0.79 | 179.53 |
| Helical rise (Å) | 4.87 | 2.44 |
|  |  |  |
| **Model refinement** |  |  |
| Model resolution (Å) | 2.4 | 2.7 |
| FSC threshold | 0.5 | 0.5 |
| Map sharpening *B* factor (Å^2^) | -49 | -43 |
| **Model composition** |  |  |
| Non-hydrogen atoms | 2910 | 1455 |
| Protein residues | 380 | 190 |
| Ligands | 0 | 0 |
| ***B* factors (Å^2^)** |  |  |
| Protein | 61 | 44 |
| **R.m.s. deviations** |  |  |
| Bond lengths (Å) | 0.008 | 0.009 |
| Bond angles (°) | 1.5 | 1.6 |
| **Validation** |  |  |
| MolProbity score | 2.0 | 2.2 |
| Clashscore | 4.1 | 5.3 |
| Poor rotamers (%) | 3.5 | 3.5 |
| **Ramachandran plot** |  |  |
| Favored (%) | 94.4 | 91.7 |
| Allowed (%) | 5.6 | 8.3 |
| Disallowed (%) | 0 | 0 |

**Table S2. Particle numbers of each data set.**

|  | All extracted particles | Type 1 | Type 2 | Type 3 | Others |
| --- | --- | --- | --- | --- | --- |
| Case1 (sarkosyl) | 211923 | 32218 | 81140 | - | 98565 |
| Case1 (aqueous) | 129112 | 27708 | 64186 | 9244 | 27974 |
| Case2 | 372553 | 17673 | 21554 | - | 333326 |
| Case3 | 259716 | 9560 | 21650 | - | 228506 |
