# Supplementary figures and images for: Cryo-EM structures of Aβ40 filaments from the leptomeninges of individuals with Alzheimer’s disease and cerebral amyloid angiopathy
Source: Acta Neuropathol Commun. 2023 Dec 4;11:191. doi: 10.1186/s40478-023-01694-8 (PMC10694933; doi:10.1186/s40478-023-01694-8)

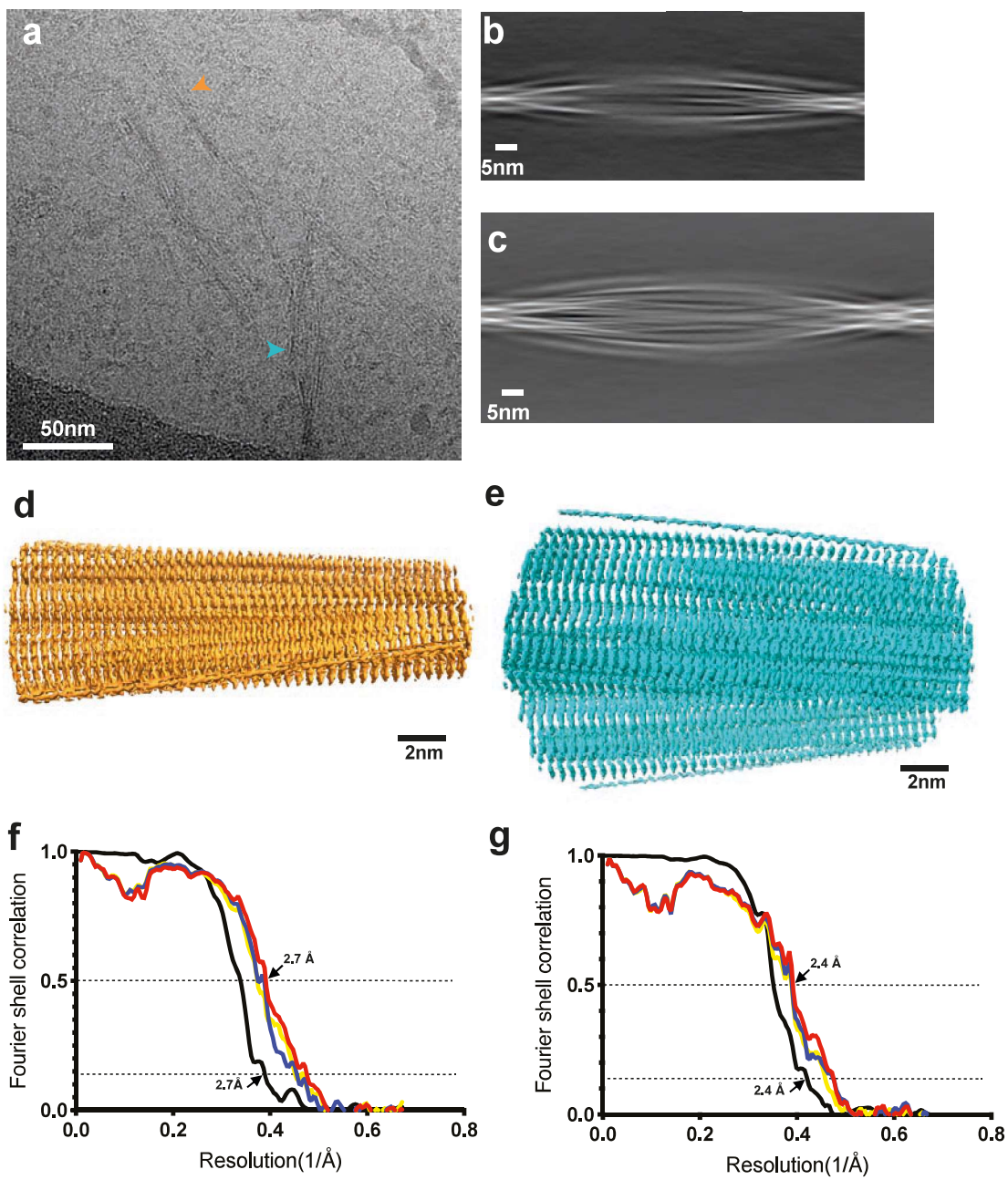

Figure S1

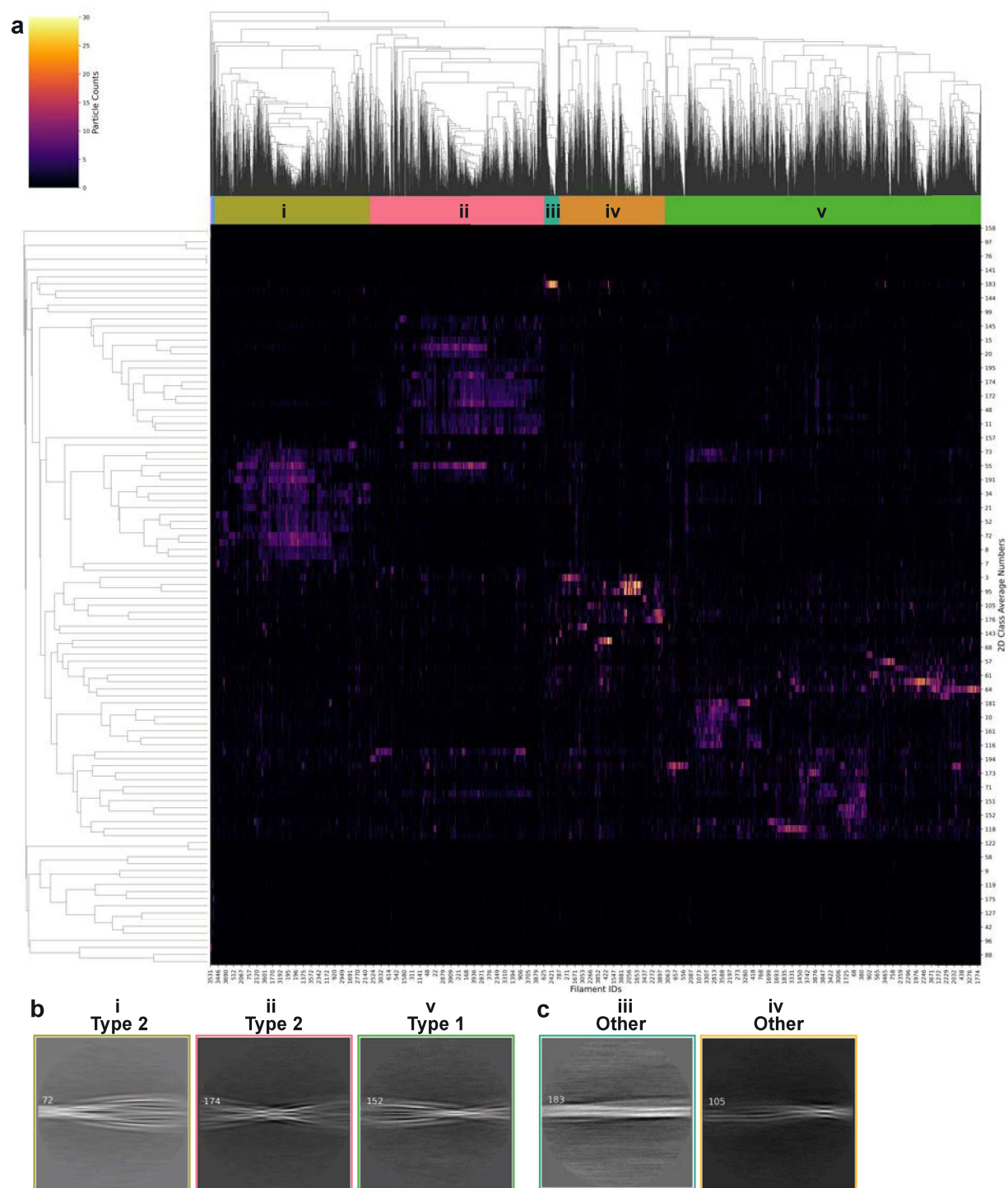

Figure S2

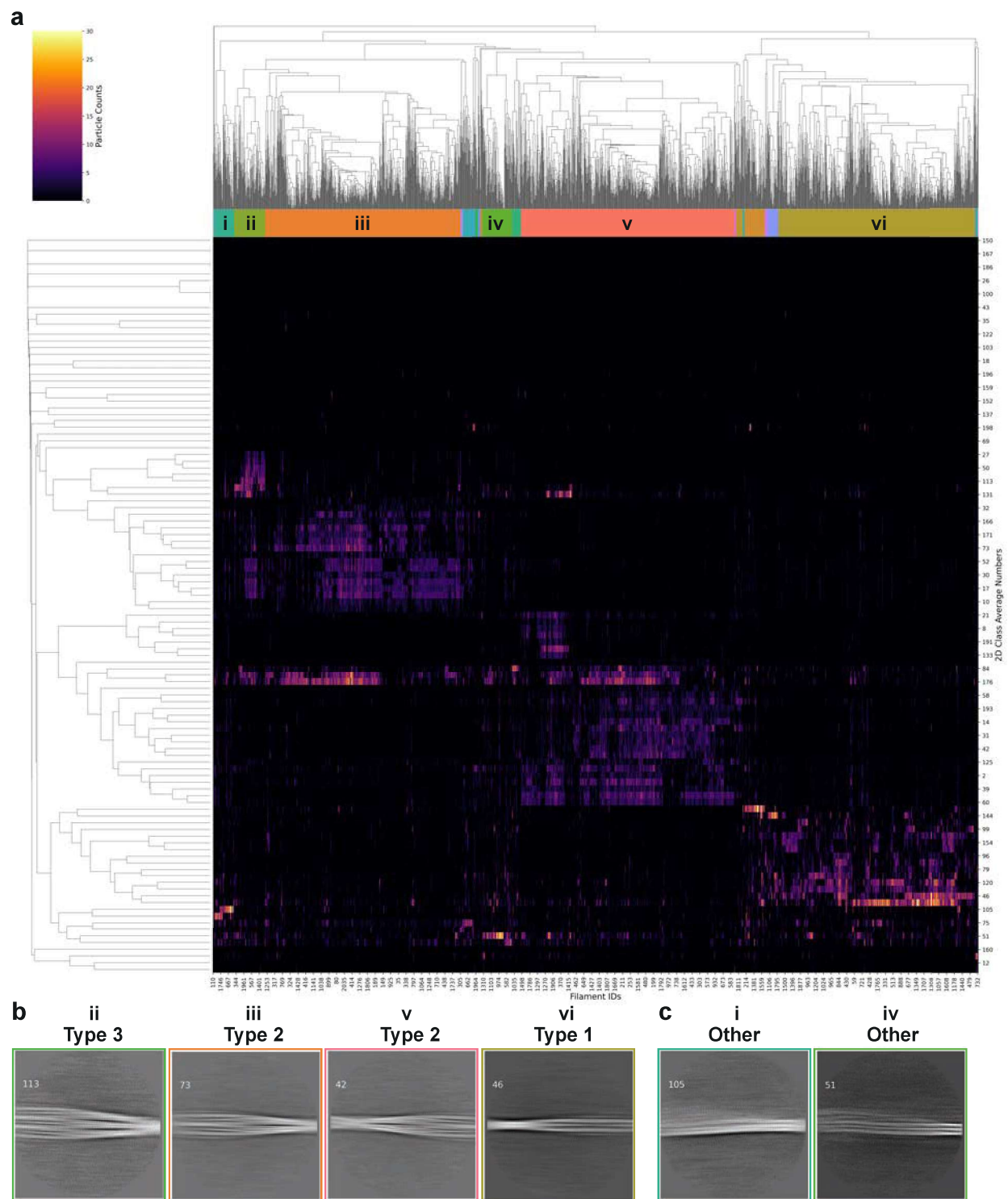

Figure S3

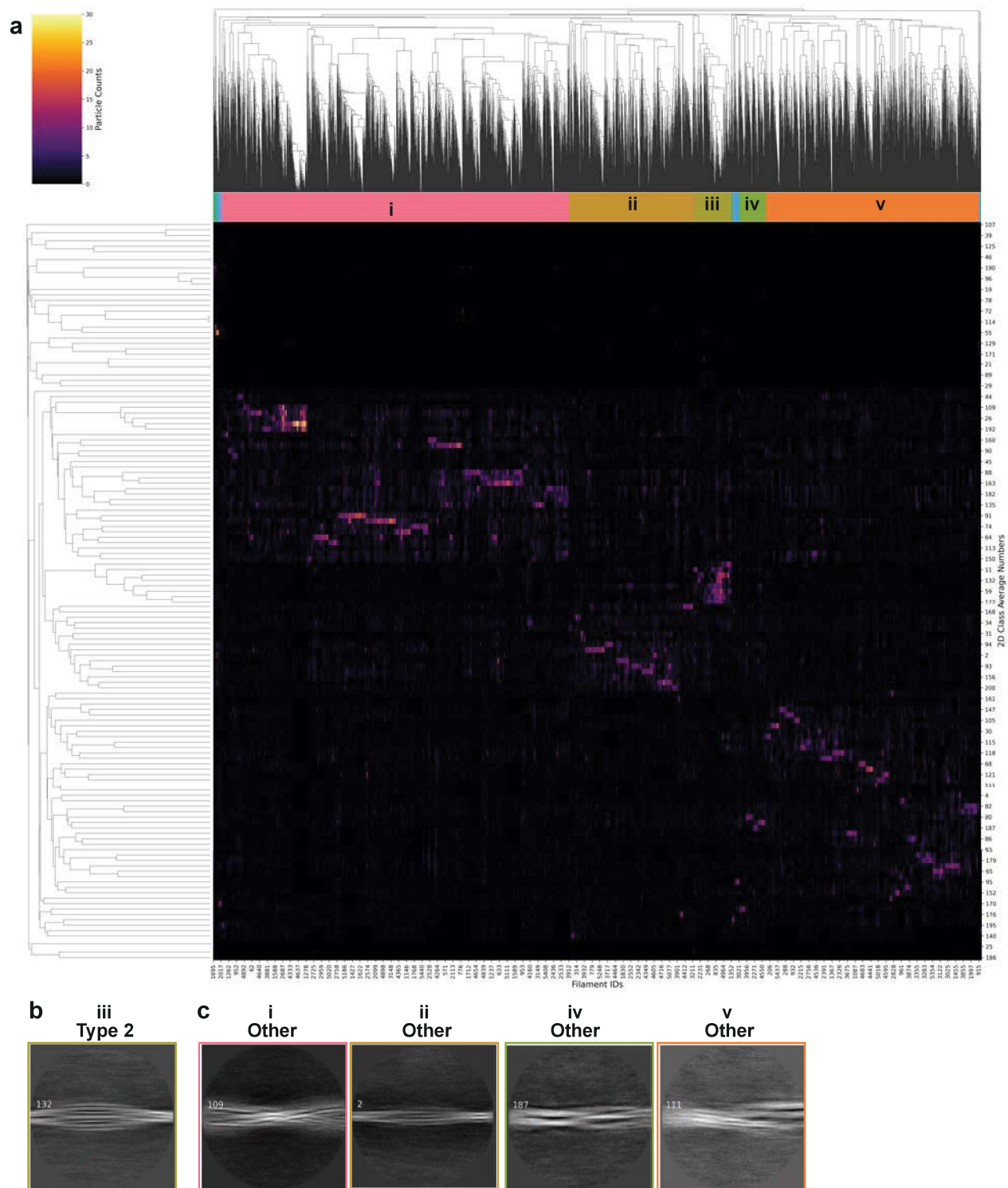

Figure S4

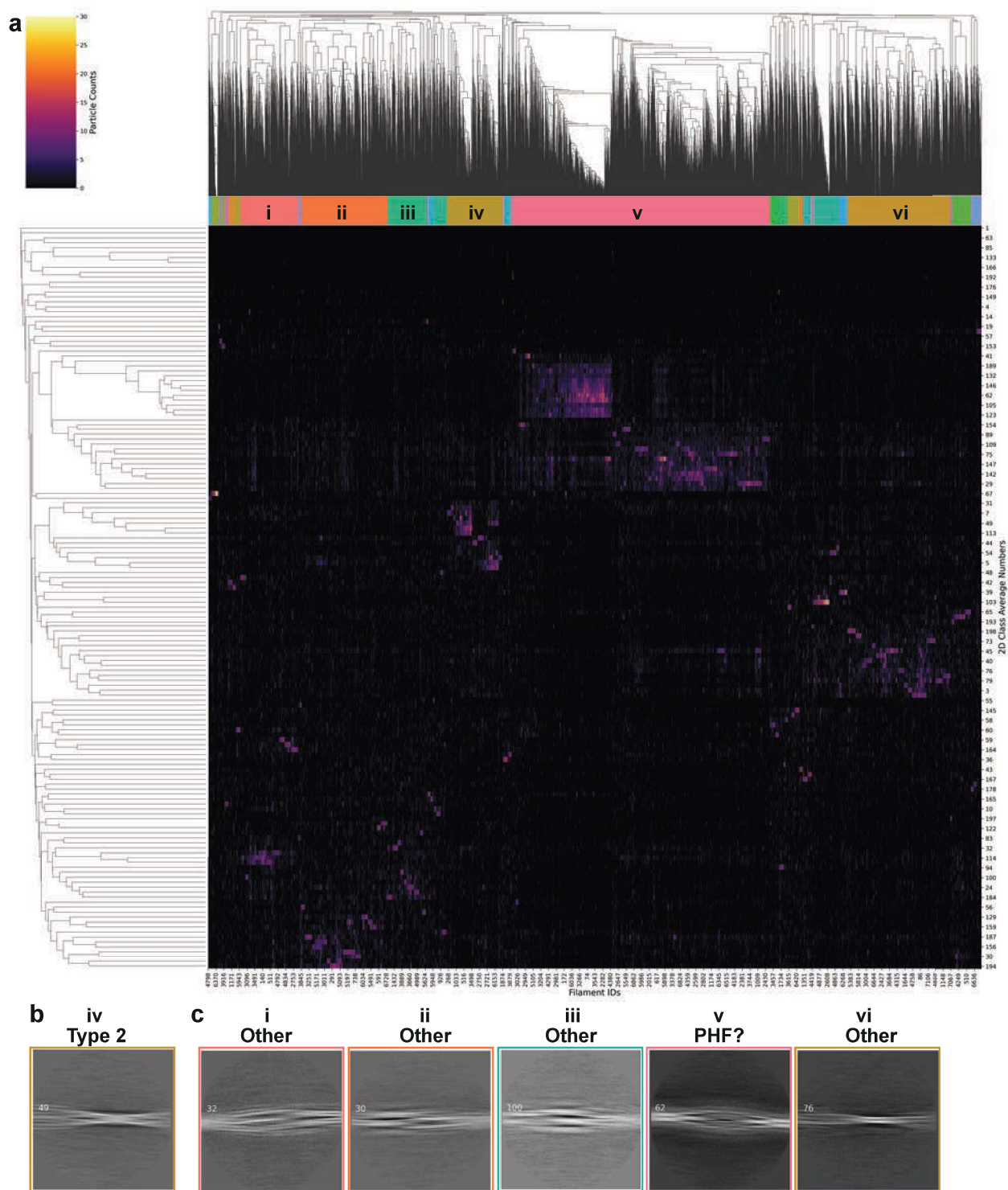

Figure S5

Supplement: Supplementary file 2 — Additional file 2: Fig. S1. Cryo-EM micrographs and processing details. a Cryo-EM micrographs of Aβ40 filaments from the leptomeninges of case 1 following sarkosyl extraction. The orange arrowhead points to a type 1 filament, whereas the cyan arrowhead points to a type 2 filament. Scale bar, 50 nm. b, c Two-dimensional class average plots of type 1 (b) and type 2 (c) Aβ40 filaments from the leptomeninges of case 1 following sarkosyl extraction. Scale bar, 5 nm. d, e Three-dimensional reconstructions of type 1 (d) and type 2 (e) Aβ40 filaments from the leptomeninges of case 1 following sarkosyl extraction. Scale bar, 2 nm. f, g Fourier shell correlation (FSC) curves for the cryo-EM maps of type 1 filaments (f) and type 2 filaments (g) are shown in black; for the refined atomic model against the cryo-EM map in red; for the atomic model refined in the first half map against that half map in blue; for the refined atomic model in the first half map against the other half map in yellow. Fig. S2. Summary of cryo-EM dataset of case 1 following sarkosyl extraction. a Hierarchical classification of individual filament segments according to their assigned two-dimensional class averages (vertical) and the picked filaments (horizontal). The segment clusters are labelled with Roman numbers. b Two-dimensional class averages of solved Aβ40 filaments. Cluster number and filament type are indicated above each panel. c Two-dimensional class averages of unsolved filaments. Cluster number and filament type are indicated above each panel. Fig. S3. Summary of cryo-EM dataset of case 1 following aqueous extraction. a Hierarchical classification of individual filament segments according to their assigned two-dimensional class averages (vertical) and the picked filaments (horizontal). The segment clusters are labelled with Roman numbers. b Two-dimensional class averages of solved Aβ40 filaments. Cluster number and filament type are indicated above each panel. c Two-dimensional class averages [file 40478_2023_1694_MOESM2_ESM.pdf]
